# Transient Receptor Potential (TRP) Channels in Pain, Neuropsychiatric Disorders, and Epilepsy

**DOI:** 10.3390/ijms24054714

**Published:** 2023-03-01

**Authors:** Felix Yang, Andy Sivils, Victoria Cegielski, Som Singh, Xiang-Ping Chu

**Affiliations:** Department of Biomedical Sciences, School of Medicine, University of Missouri Kansas City, Kansas City, MO 64108, USA

**Keywords:** transient receptor potential channels, TRP, TRPM, TRPV, TRPC, pain, depression, bipolar, epilepsy, seizure

## Abstract

Pharmacomodulation of membrane channels is an essential topic in the study of physiological conditions and disease status. Transient receptor potential (TRP) channels are one such family of nonselective cation channels that have an important influence. In mammals, TRP channels consist of seven subfamilies with a total of twenty-eight members. Evidence shows that TRP channels mediate cation transduction in neuronal signaling, but the full implication and potential therapeutic applications of this are not entirely clear. In this review, we aim to highlight several TRP channels which have been shown to mediate pain sensation, neuropsychiatric disorders, and epilepsy. Recent findings suggest that TRPM (melastatin), TRPV (vanilloid), and TRPC (canonical) are of particular relevance to these phenomena. The research reviewed in this paper validates these TRP channels as potential targets of future clinical treatment and offers patients hope for more effective care.

## 1. Introduction

**Figure 1:**
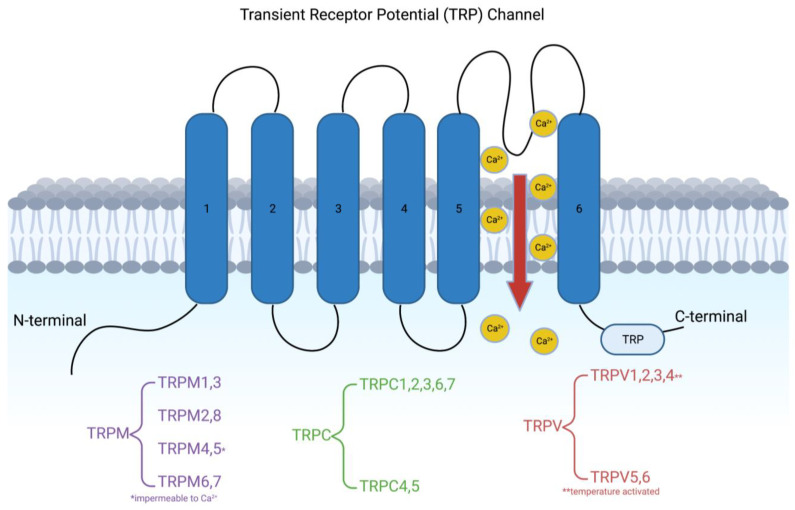
Visualization of the generic structure of the transient receptor potential (TRP) channel, consistent for most families within the class. The pore between the fifth and sixth transmembrane domains yields nonselective calcium ion permeability. TRPM4 and TRPM5 are two exceptions, as they are calcium impermeable channels (Created with BioRender.com).

The last century of scientific development, especially in neurological sciences, has emphasized describing ion channels that play various roles in pathology. Accurate descriptions can open avenues for potential treatments, alongside enabling a deeper understanding of diseases themselves. Transient receptor potential (TRP) channels are one set of those discovered that have a wealth of potential. They are a collection of proteins that mainly function as nonselective cation channels [1]. First observed in a mutant strain of Drosophila, contemporary science has delineated 28 different TRP channels across multiple species [2,3]. Looking at genetic differences, the TRP superfamily can be separated into seven different subfamilies: TRPC (canonical); TRPV (vanilloid); TRPM (melastatin); TRPP (polycystin); TRPML (mucolipin); TRPA (ankyrin); and TRPN (NOMPC-like) [4]. Each TRP family channel consists of six transmembrane helical domains (TM1-TM6), N- and C-terminal regions in the cytosol, and a loop between the TM5 and TM6 domains, forming the important channel pore (Figure 1) [5]. Each TRP is being investigated as a possible treatment target for cerebrovascular disease, pain, psychological and neurological illnesses, diabetes, and even cancer [1,6,7,8,9,10]. In this paper, emphasis is placed on TRPM, TRPC, and TRPV, concerning their roles in pain, psychiatric diseases, and epilepsy (Table 1).

Melastatin homology regions (MHRs) distinguish TRPMs from the TRP superfamily, in addition to the fact that TRPMs have the most amino acids in their systolic domain. The TPRM family can be further subdivided into TRPM1-TRPM8, where sequence homology produces related pairs as follows: TRPM1 and TRPM; TRPM2 and TRPM8; TRPM4 and TRPM5; and the last pair, TRPM6 and TRPM7 [5]. All of the TRPMs permit different ions to pass through the channel pore with some sharing similar permeability and others varying. (For an in-depth look at ion permeability, see the cited review [11]). Each of the TRPMs has a conserved Ca^2+^-binding site, but recent data suggest that only TRPM2 and TRPM8 require it for gating [11,12,13]. TRPM4 and TRPM5 are uniquely the only TRPM channels impermeable to divalent ions such as Ca^2+^, even though these are Ca^2+^-activated channels [14]. Noted mutations in genes encoding TRPMs create channelopathies that influence cancer, neuropathic pain, inflammation, hypertension, diabetes, and hypomineralization [11].

TRPC channels were given their name—TRP canonical—due to the fact they most resembled Drosophila TRP channels that were found to be responsible for light sensing [15]. There are seven members of this subfamily, TRPC1-7, which can also be further subdivided into four groups based on their sequence homology: TRPC1, TRPC2, TRPC3/6/7, and TRPC 4/5 [16]. Other studies have noted that TRPC2 is found to be a pseudogene in humans and have thus organized the subfamilies into TRPC1/4/5 and TRPC3/6/7 [17]. Mammalian TRPC channels are activated downstream from receptors that signal using phospholipase C (PLC) but are expressed in numerous tissue types with various functions that differ from similar Drosophila proteins [18]. Due to the relationship between the Ca^2+^ filling store and PLC, these channels sometimes become activated in response to changing Ca^2+^ levels and are permeable to cations, namely Na^+^ and Ca^2+^ influx [18]. Research has shown that these channels play important roles in cardiovascular and renal health, as well as being potential targets for epilepsy treatment [19,20,21,22].

TRPV1 was the first mammalian TRP channel to have its structure delineated and to be cloned [23,24]. Of this subfamily, TRPV1-4 functions as the thermal sensing channels, which are temperature-activated, while TRPV5-6 functions are not activated by temperature and instead take the more conventional TRP route of being calcium-sensitive [25]. TRPV1, 2, and 4 have been shown to influence cerebral ischemic injury where these channels demonstrate a neuroprotective effect via Ca^2+^ influx and other signaling pathways, such as JNK and p38 MAPK [26]. Further research observed TRPV1 channel activation inhibiting neutrophil infiltration and reducing free radical generation in ischemia–reperfusion injury [27]. Together, these membrane subtypes pose significant opportunities for future research and biomedical innovation.

**Table 1:** TRP Channel subfamily is involved in pain, psychiatric, and epileptic disorders.

TRP Channel Subfamily	Pain Disorders	Psychiatric Disorders	Epileptic Disorders
TRPM	TRPM2 KO reduces nocifensive response in inflammatory, neuropathic, mechanical, and thermal pain [28,29,30,31] TRPM3 facilitates heat nociceptive sensation [26,32] TRPM8 likely facilitates cold afferent sensation in context of analgesia [33,34]	TRPM2 KO precipitates bipolar behaviors [35,36] TRPM3 implicated in post-partum mood disorders, such as depression and anxiety [37,38]	TRPM2 plays a protective role against JME-associated cell death in relation to EFHC1 [39] TRPM3 overactivity associated with DEE [40,41] TRPM7 inhibition reduces seizure-induced neuronal death [42,43]
TRPV	TRPV1 inhibition resulted in increased heat pain threshold and reduced osteoarthritic pain [44,45,46] TRPV4 inhibition resulted in decreased neuropathic pain in chronic back pain models [47]	TRPV1 KO demonstrated less anxiety, freezing, and contextual fear behaviors [48,49,50,51] Increased TRPV1 expression with methamphetamine use [52]	TRPV1 agonism with capsaicin demonstrated pro-convulsant activity [53] TRPV1 antagonism with CPZ has protective role against neuronal apoptosis and epilepsy [54]
TRPC	TRPC3 likely signals both itch sensation and pain [55] TRPC5 KO had increased inflammatory joint pain but decreased touch pain in sickle cell disease, migraine, chemotherapy-related pain, and surgical pain [56]	TRPC5 implicated in transferring conditioned fear responses to amygdala [57,58] TRPC4 and 5 inhibition associated with antidepressant and anxiolytic behavioral phenotype [58,59]	Increased TRPC3 expression decreases threshold for epileptic activity [60] Increased TRPC1 expression in FCD and seen in astrocyte modulation of epilepsy [61,62] TRPC6 downregulated in chronic epileptic conditions [63]

## 2. TRPM

Table 1 has shown the TRPM in pain, psychiatric and epileptic disorders.

### 2.1. Pain Disorders

The basic mechanism of pain can be broken down into transduction, transmission, and modulation with the introduction of a noxious stimulus [64]. Furthermore, calcium ions play an important role in generating action potentials in order for nociceptive signals to be sent out through neurons [64]. Ion channels, such as voltage-gated calcium channels (VGCCs), have been well established as integral mediators of pain [65]. The role of calcium in nociception is highlighted in our review of TRP channels as several studies have portrayed this family of receptors as an important modulating target for analgesia in acute and chronic pain [66,67]. Although it has been speculated that TRPM ion channels have both pain-exacerbating and pain-alleviating mechanisms, the majority of studies using both agonism and antagonism of TRPM subtypes support their pro-inflammatory and pain-inducing functions [28,29]. 

TRPM2 channels are one such family of proteins that have interactions with reactive oxidative species (ROS) [30] and is involved in pathological pain [31]. The current understanding of the receptor is that it acts as a sensor for both ROS and adenosine diphosphate ribose (ADPR) in calcium gating [31]. Its expression in mice is mostly seen in primary afferent sensory neurons, including both A and C fiber neurons [31]. TRPM2 knock-out (KO) mice in a study conducted by Haraguchi et al. demonstrated reduced nocifensive response in inflammatory, neuropathic, mechanical, and thermal pain, without the loss of baseline sensitivity to mechanical and thermal sensation [32]. Primarily expressed in somatosensory dorsal root ganglion (DRG) neurons, thermogenic TRPM3 plays an integral role in heat nociception. TRPM3 knockout mice displayed a decreased response to noxious heat stimuli [26,68]. Blockage of the receptor by hesperetin, isosakuranetin, and primidone decreased intracellular transport of calcium ions, and similar to TRPM3 knockout mice, rodents treated with flavanones and primadone displayed decreased pain behaviors in response to noxious heat stimuli [33].

The development of the antagonists and modulators of TRPM channels is already underway, especially for TRPM8. TRPM8 was first characterized as a receptor for cold sensory afferents, which are activated by certain natural molecules known to produce this sensation, such as menthol, icilin, and eucalyptol [34]. The goal of cold sensation in the context of this receptor’s activation is the promotion of analgesia. However, TRPM8 is also believed to be involved in pain amplification due to cold hypersensitivity. While more research must be conducted to identify a definitive role in thermal-associated pain, knockout mice of this gene lacked cold hypersensitivity [69]. Other implications of pain for TRPM8 include migraines and bladder pain [70,71,72]. It is likely that TRPM8 is expressed for cold-afferent functions, and the modulation of the receptor shows potential for thermoreceptive pain analgesia.

### 2.2. Psychiatric Disorders

Unfortunately, most psychiatric illnesses are without complete descriptions of their etiology. One new theory contends that the oxidative state of cells and subsequent downstream calcium changes may have an influence [73]. While it is highly contested whether the antioxidant system is upregulated or downregulated in psychiatric disorders, many studies demonstrate changes in superoxide dismutases (SODs), catalase (CAT), and glutathione peroxidases (GPXs) that correlate with mental health issues [73,74]. A meta-analysis of double-blind, randomized, placebo-controlled trials evaluating the effects of N-acetyl cysteine (NAC) found that treatment significantly improved depressive symptoms in heavy smokers, those with trichotillomania, and those with bipolar or depressive disorders [73,75]. One double-blind, randomized, placebo-controlled, clinical trial of its efficacy as an adjunctive treatment for patients with schizophrenia on antipsychotic medications showed that patients taking NAC presented improvements on the Positive and Negative Syndrome Scale (PANSS) compared to the placebo group [76]. The same trial demonstrated significantly improved cognitive functions [76]. However, how exactly do TRPM channels relate to the psychiatric significance of redox reactions at the cellular level?

The TRPM2 channel is among a group of highly sensitive proteins that are influenced by redox status [73]. When they are activated, they induce Ca^2+^ entry and transfer redox activity into intracellular Ca^2+^ signaling [73]. Specific details have been more thoroughly reviewed elsewhere [77]. With only this information, one can see the theoretic bridge between redox reactions, intracellular calcium, and psychiatric illness that TRPM channels provide. Two studies have demonstrated the need for TRPM2 in order to have NMDAR-dependent long-term depression in the hippocampus [78,79]. Another investigation demonstrated the key role that TRPM2 channels play in the H_2_O_2_-dependent modulation of substantia nigra pars reticulata GABAergic neurons [35]. 

Fine mapping linkage analysis showed that TRPM2 on chromosome 21q is associated with bipolar disorder [36]. One of the identified single nucleotide polymorphisms (SNPs) leads to a deletion of TRPM2, and subsequent studies demonstrated that TRPM2 KO mice exhibit bipolar-disorder-related behaviors [37]. What is even more intriguing is that one of the essential targets of lithium is GSK-3, which experiences increased phosphorylation whenever TRPM2 is disrupted [37,79]. Other findings expand the likely effects of TRPM2 channels to include major depressive disorders, as well as other behavioral phenotypes that are related to oxidative stress [38]. Beyond TRPM2, recent research highlights TRPM3 as a potential player in the development of mood and anxiety disorders, with specific mention of post-partum mood disorders [80]. Similar efforts revealed that TRPM3 expression was changed in a mouse model of bipolar disorder, furthering the aforementioned evidence [81]. 

### 2.3. Epileptic Disorders

Epilepsy is a chronic neurological disorder caused by abnormal electrical excitability in the brain. Calcium ion (Ca^2+^) accumulation has been hypothesized to play a critical role in the etiology of this disease [82]. Though not fully understood, the activation of tissue transglutaminase (tTG) by calcium accumulation is thought to trigger glutamate-induced neurotoxicity that yields seizure activity [83]. This hypothesis is like that of neuronal injury in brain ischemia and trauma, where the amount of calcium influx correlates with the degree of damage [84]. Loss-of-function K^+^ channels and gain-of-function Na^+^ channels have also been identified as contributors to neuron excitability and causes of epilepsy. TRP channels are nonselective transmembrane cation proteins that allow cations such as Ca^2+^ to pass through their pores [39,85,86]. Because ionic disbalance plays a central role in the etiology of epilepsy, these nonselective TRP channels serve as areas of interest within the field. 

Three TRPM channels that have shown direct effects in epilepsy include TRPM2, TRPM3, and TRPM7. TRPM2 is co-expressed and has direct physical interactions with the EF-hand motif-containing protein, EFHC1. Typically, EFHC1 enhances the susceptibility of TRPM2 to neuronal apoptosis through ROS and H_2_O_2_. In juvenile myoclonic epilepsy (JME), however, there is a mutation in EFHC1. Given the direct relationship between EFHC1 and TRPM2, it is suggested that TRPM2 plays a protective role against cell death that contributes to the phenotypic presentation of JME [40]. 

Developmental and epileptic encephalopathies (DEEs) are groups of conditions characterized by epilepsy with comorbid developmental delay. Gain-of-function mutations that cause overactivity in the TRPM3 channel are associated with DEEs [41]. Like most other TPR channels, TRPM3 is a nonselective cation channel that is permeable to calcium [42]. Primidone, a clinically approved antiepileptic, is a TRPM3 antagonist whose antiepileptic effects could be in part due to its inhibition of TRPM3 [41]. Therefore, the development of TRPM3 inhibitors serves as a focus for future epileptic therapeutics. 

Within the brain, TRPM7 is activated and expressed during epilepsy, perpetuating a positive feedback loop and contributing to the production of ROS. This is confirmed by the fact that TRPM7 ablation prevents a cation current under circumstances of oxygen–glucose deprivation, and therefore prevents ROS-mediated cell death [43]. A recent study published in the International Journal of Molecular Sciences demonstrated that TRPM7 inhibition reduced the seizure-induced expression of TRPM7 channels [87]. This resulted in decreased intracellular zinc accumulation, ROS production, and postictal apoptotic neuronal death [87].

Other TRPM channels have demonstrated regulatory roles that could translate to the pathogenesis of epilepsy if found within epileptic regions of the brain. GTL-2 is a TRPM that influences local ion composition in non-neuronal tissues. Its function in the epidermis has been compared to the modulatory role of glial cells, as GTL-2 channels contribute to ionic buffering and glutamate uptake that regulate neurotransmitter release [88]. If found in neuronal tissues, GTL-2 could play a role in the ionic modulation of Ca^2+^ that yields glutamate release during seizures. In the mouse retina model, TRPM1 localized to the dendrites of ON-bipolar cells and modulated depolarization in response to light-induced glutamate release [89]. Similar to GTL-2, if TRPM1 contributes to depolarization and glutamate release in brain regions impacted during epileptic episodes, its inhibition could hold a significant role in regulating seizures. 

## 3. TRPV

Table 1 also has shown the TRPV in pain, psychiatric and epileptic disorders.

### 3.1. Pain Disorders

Among the TRP channels, ones belonging to the TRPV family appear to have the strongest backing from the literature, supporting their roles in modulating pain sensation. TRPV1 is expressed in a variety of tissues, including sensory ganglia and small sensory neural fibers [90]. More than a decade ago, early rodent models using TRPV1 antagonism highlighted the receptor’s role in thermosensation and pain perception, similar to that of TRPM [44]. This idea was further tested in human trials, where TRPV1 antagonist SB-705498 produced significant results in increasing the heat pain threshold in treated skin [45].

More recently, one study reported inhibition of pain behavior in osteoarthritis (OA) mice after intra-articular administration of JNJ-17203212, a TRPV1 antagonist [46]. This was measured by evaluating the attenuation of weight-bearing asymmetry after antagonism of the receptor [46]. The same study also found that there was an increased expression of this receptor in human OA, rheumatoid arthritis (RA), and postmortem (PM) synovium via immunohistochemistry [46]. Based on this evidence, modulation of TRPV1 may prove to be beneficial in attenuating inflammatory joint pain, especially because TRPV1 has been seen to respond to oral paracetamol antinociception in human patients [91]. In addition to joint pain, TRPV1 expression in microglia in anterior cingulate cortices, GABAergic spinal interneurons, as well as other sensory ganglia, plays roles in numerous pain mechanisms of neuroinflammation, allodynia, and mechanical hyperalgesia [92,93,94]. Interestingly, cannabidiol (CBD) is another molecule that has been tested to modulate TRPV1 to observe its role in analgesic pathways. Cannabis-based products have become easily accessible and clinical trials in humans have supported their usage in self-medicated pain relief [29,95]. In a Parkinson’s disease mouse model, CBD increased anandamide binding to TRPV1 receptors and produced analgesic effects [96]. CBD’s exertion of pain alleviation may be dose-dependent as one group found that concentrations of 10 and 50 μmol/L CBD were required for decreased calcium shuttling in TRPV1 channels [47]. 

The TRPV4 receptor is expressed in a variety of tissues, including immune cells, sensory neurons, glial cells, the spinal cord, cortical pyramidal neurons, the thalamus, and cerebellum basal nuclei [97]. Similar to TRPV1, TRPV4 is thermosensitive and can be activated by heat, as well as by mechanical forces [97]. One of the most important roles of TRPV4 found in studies is the alleviation of neuropathic pain via its inhibition. A major source of chronic pain commonly seen in patients is chronic back pain. In several studies, TRPV4 antagonism was seen to reduce cartilage degradation after mechanical compression, reduce IL-1β-mediated NO release, and decrease neuropathic pain in chronic compression of DRG pain models in rats [98,99,100]. One pre-clinical candidate thought to inhibit TRPV4, named GSK3527497, was described to be a potential TRPV4 antagonist that can be used for therapeutic pain management [101]. More recently, GSK3527497 generated favorable results and was found to be well tolerated in Phase I clinical trials in both healthy volunteers and heart failure patients after 14 days of dosing [57]. While more studies utilizing human subjects are needed, these findings suggest antagonists of TRPV4 could be a new type of pharmacotherapy in the future. 

### 3.2. Psychiatric Disorders

As mentioned in the section discussing TRPMs, TRP channels are suggested to play a role in the etiology of psychiatric illnesses. In the TRPV family, TRPV1 has amassed the most evidence, indicating its relevance in the realm of mental health [48]. For example, TRPV1 KO mice have shown less anxiety-related behavior, freezing, and contextual fear in different methodological designs [49]. These findings were paralleled by an impairment in hippocampus-dependent learning, consisting of a deficit in long-term potentiation, not unlike the TRPM2 findings [49]. Further investigations have examined the role of these physiological manipulations in the etiologies of mental disorders. 

One study looked at the antidepressant effects of TRPV1 agonists and found that the administration of capsaicin and olvanil provided significant protective effects against induced depression in mice [50]. Another experiment focusing on the ventral hippocampus in rats found that TRPV1 channels have an important role in regulating anxiety [51]. This was shown via the antagonism of the TRPV1 channels via capsazepine and was redemonstrated by Kasckow et al. [50]. Outside of the hippocampus, similar findings have been observed in the periaqueductal grey where, again, antagonism of TRPV1 led to anxiolytic-like effects [52]. Another set of mental health issues, namely substance use disorders, are also found to correlate with TRPV1 activity. Multiple studies demonstrate that continued methamphetamine exposure leads to increases in TRPV1 expression, specifically in the frontal cortex [102]. Another investigation found that the deletion of TRPV1 channels led to an altered behavioral response to ethanol administration in mice [103]. Together, these findings demonstrate that TRPV1 is a significant target for future research regarding the treatment of psychiatric diseases. 

### 3.3. Epileptic Disorders

TRPV1 is one of the most well-characterized and documented transient-receptor potential-vanilloid channels in the TRPV family. Like other TRP channels, it yields nonselective Ca^2+^ permeability. TRPV1 was initially found to be highly expressed in the DRG, the sensory neurons. This paved the way for the discovery of the significant role of TRPV1 in inflammatory hyperalgesia [24]. Subsequent research demonstrated that TRPV1 is also located in various nuclei locations within the human brain, including the cortex, hypothalamus, and hippocampus [53]. Glutamate excitation through Ca^2+^ activation, especially in the calcium-sensitive hippocampus, has long been an important etiologic consideration for epilepsy [39]. The function of TRPV1 in hippocampal excitation thereby highlights a new focus in the field. 

Capsaicin is a TRPV1 agonist that can be used to elicit the effects of the channel. In the mouse model, capsaicin demonstrated pro-convulsant activity that was only blocked by pretreatment with capsazepine (CPZ), an antagonist of TRPV1 [104]. Further, systemic or hippocampal administration of a TRPV1 antagonist reduced the susceptibility of mice to pentylenetetrazol (PTZ)-induced seizures [54]. The activation of TRPV1 by capsaicin caused apoptosis in the hippocampi and DRG of rats, indicating that this channel is important for regulating epileptic episodes. Conversely, TRPV1 blockers such as CPZ demonstrated a protective role against neuronal apoptosis and epilepsy in the same rat model [105]. Cannabidiol (CBD) is argued to be an antagonist and agonist of TRPV1 and TRPV2, respectively [47,106]. In either case, CBD administration decreased in vitro epileptiform activity and in vivo seizure activity in rats [107]. In a descriptive study of humans, increased expression of TPRV1 mRNA/proteins was found in patients with mesial temporal lobe epilepsy compared to the control [55]. These findings provide evidence of the role of TRPV1 functions in perpetuating seizures among animals and likely humans. Future work concentrating on TRPV1 inhibitors as antiepileptic therapeutics remains a promising area to be researched.

## 4. TRPC

Table 1 has shown the TRPC in pain, psychiatric and epileptic disorders.

### 4.1. Pain Disorders

All TRPC channels reveal calcium permeability, which is an important part of pain transduction and sensitization of nociceptors as previously mentioned. Based on this cellular mechanism, it is worthwhile to continue the investigation of the TRPC channels’ role. While new research surrounding TRPC3 leans more toward its role in non-histaminergic itch, it is likely that TRPC3 signals both itch and pain, given that it is expressed heterogeneously in both nociceptors and pruriceptors [108]. In situ hybridization in one study found that TRPC3 was exclusively expressed in small-to-medium-diameter sensory neurons in rat DRG [109,110]. Disruption of endogenous TRPC3 in these ganglia resulted in decreased expression of store-operated calcium entry (SOCE), UTP, or protease-activated receptor 2 (PAR2) agonist-evoked calcium transduction [109]. TRPC3′s contribution to calcium-induced nociceptor sensitization and its relationship with co-expression with other proinflammatory receptors support its importance in future pain therapies. The strongest support for the future of TRPC-related non-opioid analgesia comes from research surrounding TRPC5. Recently, the function of this receptor in pain perception was observed in murine models of osteoarthritis where TRPC5 KO mice had significantly exacerbated pain-like behaviors compared to that of the wild type (WT) [111]. Along with behavioral results, findings were also found at the microscopic level where TRPC5 KO mice had increased mast cell markers and extracellular matrix remodeling, synonymous with synovial inflammation [111]. One group shared similar findings with TRPC5 gene-deleted mice and saw increased weight-bearing asymmetry, inflammatory cytokines, and secondary hyperalgesia in human inflammatory arthritis synovia after chronic treatment of a TRPC5 antagonist (ML204) [56]. While some osteoarthritis models suggest TRPC5 inhibition leads to more inflammation and pain, other researchers saw reversed touch pain in mice models of sickle cell disease, migraine, chemotherapy-related pain, and surgical pain after TRPC5 inhibition [58]. TRPC5 appears to have great translation potential as well, due to its high expression in donor human DRG tissues [58].

### 4.2. Psychiatric Disorders

Of all the TRP channels, TRPC channels have become the biggest target for potential treatments [48]. For one, TRPC5 is thought to be responsible for transferring conditioned fear responses to the amygdala, as they are expressed in the thalamus, amygdala, and cortical areas related to fear responses [48,112]. TRPC4 was identified by the same group of researchers to be essential for behavioral responses to anxiety-inducing stimuli, seeing as KO mice demonstrated an anxiolytic behavioral phenotype [112]. Because TRPCs are expressed in B lymphocytes, one study found that expression on those cells was changed in those with bipolar disorder, suggesting a functional change in the channel in the disease state [113]. Interestingly, treatment for 7 days with lithium reduced the TRPC3 protein in those same cells in bipolar patients [114]. 

Looking more at the impact on neuronal populations implicated in the reward system and other goal-oriented behavior, one study found that TRPC1 deletion led to a loss in striatal cells, in addition to proteomic alterations [115]. A further physiological mystery of the TRPC channels in the brain was found in an investigation of IL-10 KO mice. These mice had reduced TRPC5 expression in the medial prefrontal cortex and amygdala [59]. These mice then demonstrated enhanced depressive and anxiety-like behavior, potentially relating the immune system to the TRPC channels already implicated in depression and anxiety [59]. Some researchers have taken these TRPC findings far enough to develop treatments with novel agents. 

In fact, acute treatment with a novel TRPC4/5 inhibitor was found to produce antidepressant and anxiolytic effects in mice [116]. Furthermore, they posit that these effects are mediated through downstream signaling involving BDNF [116]. Another study with a different but equally novel TRPC4/5 inhibitor was able to generate similar findings [60]. The compound reduces CCK-4-invoked neuronal activity in amygdala slices, the brain region known to produce fear responses [60]. Together, these findings suggest once more that TRP channels are implicated in psychiatric illness and physiology, specifically the TRPC channels mentioned above.

### 4.3. Epileptic Disorders

Among the TRPC families, TRPC3 is the most reviewed within the setting of epilepsy. While all TRP channels regulate cations in some regard, TRPC3 uniquely mediates low Mg^2+^ and Ca^2+^ depolarization contributing to epilepsy. In addition, higher expression of TRPC3 makes the immature cortex more excitable and thus decreases the threshold for epileptic susceptibility [117]. In animal models of pilocarpine-induced status epilepticus, TRPC3 has been studied using different pharmacologic agents. The main outcomes from targeting this channel with Pyr3, a TRPC3 inhibitor, included decreases in TRPC3 channel expression and reductions in the root mean square of power/theta activity of seizures [118]. In hyperthermia-induced febrile seizure rats, elevated mRNA and protein levels were detected in neuronal cells within the hippocampus. Accordingly, when the Pyr3 inhibitor was administered in this model, seizure severity, neuroinflammation, and neuronal cell death decreased [119]. While Pyr3 is a selective and potent TRPC3 inhibitor, its metabolic instability and toxicity limit its full benefit. A modified pyrazole compound, JW-65, was recently studied for its effects on TRPC3. Systemic administration of JW-65 prior to pilocarpine induction showcased a delay in seizure initiation. JW-65 administration after pilocarpine-induced seizures initiated anti-seizure activity, confirmed by electroencephalographic monitoring with video. Seizure susceptibility decreased in a dose-dependent manner in accordance with JW-65 administration [61]. These findings suggest that JW-65 is a novel therapeutic of interest in seizure attenuation. In conjunction, the results of these TRPC3 studies demonstrate that TRPC3 is important in promoting electrical excitability within various seizure models. Inhibition of this channel proves significant to reducing seizure activity. Thus, TRPC3 channel inhibitors serve as an area of interest in the field of anticonvulsant therapeutics. 

TRPC1 and TRPC6 are two other TRPC channels of epileptogenic interest [62]. Among humans with focal cortical dysplasia (FCD), increased mRNA and protein expressions of TRPC1 were found in cortical tissue. Such findings of overexpression in cell-specific patterns suggest that TRPC1 may play a role in creating conditions permissive for FCD. Notably, TRPC1 also showed colocalization with GFAP in reactive astrocytes, indicating that the channel may aid in astrocyte modulation of epilepsy [63]. 

TRPC6 holds unique consideration in epilepsy, as it was shown to be downregulated under chronic epileptic conditions in the rat model. Furthermore, ablation of TRPC6 by protein-specific silencing RNA causes increased susceptibility to pilocarpine-induced seizures. This may be because TRPC6 is involved with signaling the expression of proteins important to mitochondrial function [120]. A deeper understanding of TRPC6 in neuroprotective signaling pathways will be beneficial for understanding the impact of this channel, especially in the setting of epilepsy.

TRP channel subfamily involved in pain, psychiatric, and epileptic disorders have been shown in Table 1.

## 5. Conclusions

TRP channels are a group of multifunctional membrane proteins that serve important functions in neurological physiology and pathology. They significantly influence oxidative species sensing, afferent nociceptive signaling, bipolar disease, depression, anxiety, and seizure disorder [35,39,66,67,78,121,122]. In addition, TRP channels play important roles in cardiovascular and renal health [19,20]. Most TRPs highlighted in this review are subjects of pharmacological development and intense study, especially to mitigate the progression of these pathological conditions. The aim of this review was to examine TRPM, TRPV, and TRPC channels and gain perspective and understanding of their growing impact on pain sensation, psychiatric diseases, and epilepsy.

Regarding pain, TRP channels represent an area of focus that is of high interest to researchers and pharmaceutical companies to develop non-opioid interventions for chronic pain. Currently, medical management of chronic pain remains dominated mainly by opioid analgesics, but the downsides of addiction and tolerance make it an unsatisfactory long-term treatment option for pain [123,124]. Thus, advancements in modulating TRP channels and nociceptive signaling from the peripheral nervous system are a more favorable route. TRPM8 is implicated in cold hypersensitivity and is theorized to alleviate inflammatory or neuropathic pain, but there is still controversy regarding the effects of TRPM8 agonism and antagonism on pain perception [34,69,125]. Inhibition of TRPV1 and TRPV4 is related to several pain-alleviating findings, especially neuropathic pain and mechanical hyperalgesia seen in inflammatory joint disease [46,91,92,93,94]. TRPV1 is also believed to serve a first-line defense role in noxious heat stimuli via capsaicin [126]. A growing field of pharmacological therapeutics that target TRPV1 includes CBD, which decreases Ca^2+^ transduction in DRG after stimulation with capsaicin [29,47,95]. In the setting of chronic neuropathic pain, non-opioid antagonism of TRPV4 shows promising data to be a major analgesic target [57]. While TRPC3 shares similar characteristics to the previously mentioned properties of TRPs, TRPC5′s physiological role likely leans more toward reducing inflammation and pain [56,108,111].

Although several psychiatric diseases do not have completely clear mechanisms of pathology, a wealth of research support TRP channel expression and function in the setting of neuropsychiatric diseases. In fine-mapping linkage of bipolar subjects, TRPM on chromosome 21q is seen to be associated with bipolar disease behaviors [36,37]. While lithium is traditionally used for bipolar symptoms, TRPM2 is a candidate for several new compounds developed within the last 5 years. Namely, ADPR analog TRPM2 antagonists may prove beneficial in the future for a spectrum of neuropsychiatric diseases, including bipolar disease [127]. There is literature supporting TRPV1′s antagonism benefiting anxiety, and its agonism promoting protection against depression, anxiety, and substance use disorder [49,51,102,103,128,129]. Antagonism of TRPV1 is a possible route for the reduction in anxiety and fear responses with future translational research [49,129]. Continuing the mechanisms of anxiety, TRPC4 and TRPC5 are heavily implicated in the transmission of conditioned fear responses to the amygdala, and gene disruption in mice demonstrated anxiolytic effects [48,112]. Two antagonists of TRPC4/5, M084 and HC-070, have already been developed, and researchers found that the administration of these two agents induced anxiolytic and anti-depressant effects in mice [60,116]. Together, these findings highlight TRPC’s important role in the physiology of anxiety and depression.

Cation transduction, including that of calcium ions, is believed to play a massive role in the pathology of epilepsy and seizures, possibly due to the glutamate-induced neurotoxicity from tissue transglutaminase activation [82,83,130]. Because ion imbalances serve a central role in the etiology of epilepsy, TRP channels are a major area of interest in understanding and treating epilepsy [82,85,86]. TRPM2 may be a channel protein of interest in juvenile myoclonic epilepsy as EFHC1, the candidate gene for this disease, potentiates TRPM2 in ROS-mediated neuronal death [40]. Primidone, along with diclofenac and maprotiline, have all been demonstrated to be efficient blockers of TRPM3 with IC50 at 0.6–6 μM [131]. Other TRPM channels that have the potential to be therapeutic targets in epilepsy include TRPM7, where inhibition reduced the seizure-induced expression of TRPM7 channels and reduced ROS-related neuronal death [43]. Agonism of TRPV1, which is expressed in the brain, hypothalamus, and hippocampus, with capsaicin and pentylenetetrazol (PTZ) demonstrated pro-convulsant activity that was only blocked with TRPV1 antagonism [54,104,126,132]. Microscopically, researchers saw apoptotic mechanisms in hippocampal and DRG cells of rats after capsaicin TRPV1 agonism, which strengthens its relationship with seizure etiology [105]. The administration of CBD also decreased in vitro and in vivo seizure activity in rat [107]. Its direct effects on TRPV1 and TRPV2 is not certain as it more strongly modulates cannabinoid receptors. Expression of several TRPC channels is consistently seen to have an impact on chronic epileptic conditions, and TRPC3 uniquely mediates low Mg^2+^ and Ca^2+^ depolarization, contributing to epilepsy [61,62,63,117,118,120].

There is abundant information and research highlighting TRP channels and their roles in pathological processes involving cation transduction through cellular membranes. Clinical trials regarding TRP modulation are already undergoing and have generated promising results. Future investigations into pharmacological interventions of TRP channels will propagate further developments in patient treatments, especially in the setting of pain, neuropsychiatric diseases, and epilepsy.

## Data Availability

Not applicable.
